# Supplementary material for: Mutations in PCYT2 disrupt etherlipid biosynthesis and cause a complex hereditary spastic paraplegia
Source: Brain. 2019 Oct 22;142(11):3382–97. doi: 10.1093/brain/awz291 (PMC6821184; doi:10.1093/brain/awz291)
Supplement: awz291_Supplementary_Data [file awz291_supplementary_data.zip › awz291-Suppl_data/Supplementary_Data4.zip!/Supplementary information 3 - fibroblast lipidomics.zip!/Fibroblast lipidomics/Patient1 vs Patient2/Statistical plots and data/Heatmap sorted on p-value and FC.pdf]

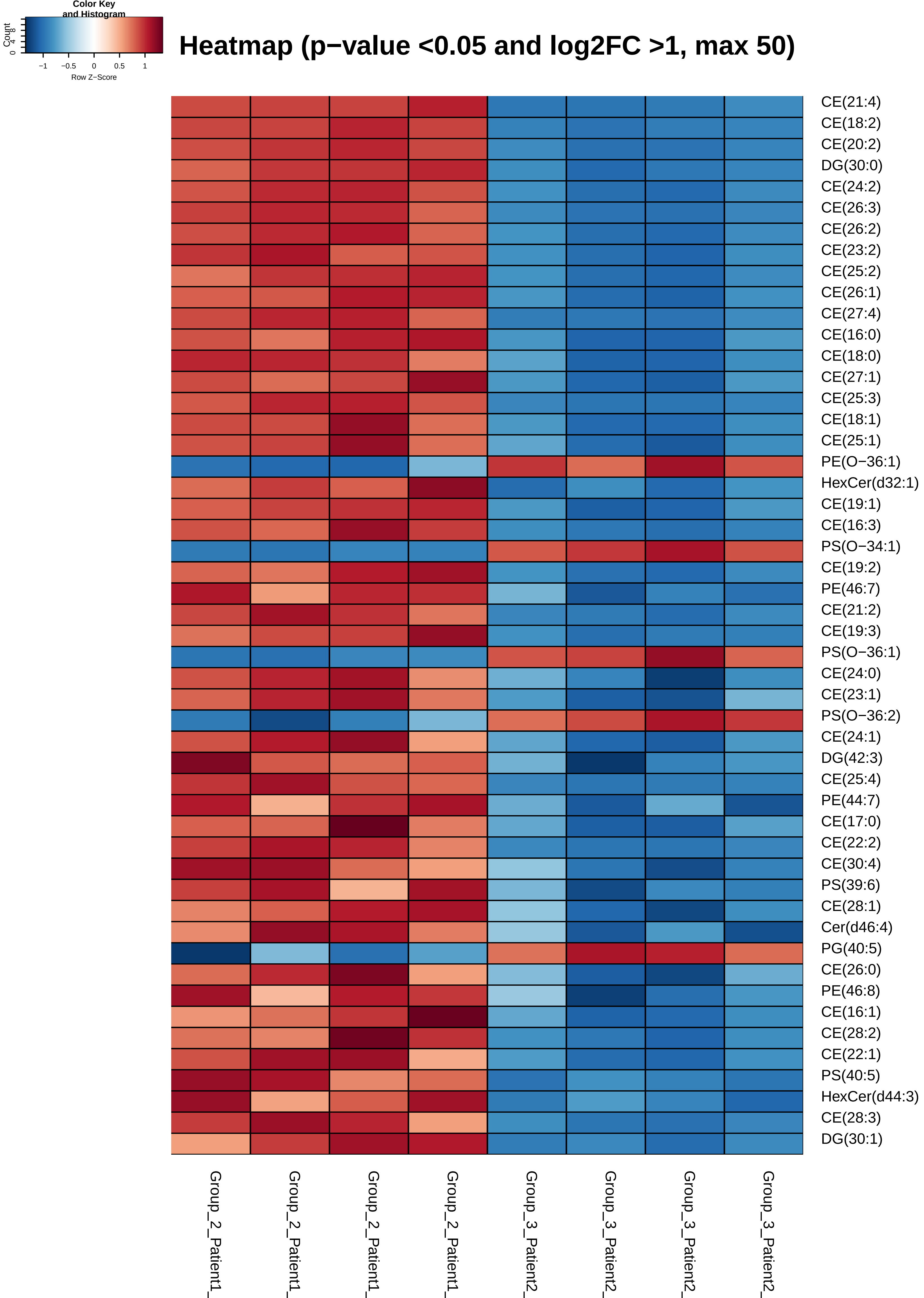
Color Key
and Histogram
Count
0
4
8
−1
−0.5
0
0.5
1
Row Z−Score
Heatmap (p−value <0.05 and log2FC >1, max 50)
CE(21:4)
CE(18:2)
CE(20:2)
DG(30:0)
CE(24:2)
CE(26:3)
CE(26:2)
CE(23:2)
CE(25:2)
CE(26:1)
CE(27:4)
CE(16:0)
CE(18:0)
CE(27:1)
CE(25:3)
CE(18:1)
CE(25:1)
PE(O−36:1)
HexCer(d32:1)
CE(19:1)
CE(16:3)
PS(O−34:1)
CE(19:2)
PE(46:7)
CE(21:2)
CE(19:3)
PS(O−36:1)
CE(24:0)
CE(23:1)
PS(O−36:2)
CE(24:1)
DG(42:3)
CE(25:4)
PE(44:7)
CE(17:0)
CE(22:2)
CE(30:4)
PS(39:6)
CE(28:1)
Cer(d46:4)
PG(40:5)
CE(26:0)
PE(46:8)
CE(16:1)
CE(28:2)
CE(22:1)
PS(40:5)
HexCer(d44:3)
CE(28:3)
DG(30:1)
Group_2_Patient1_
Group_2_Patient1_
Group_2_Patient1_
Group_2_Patient1_
Group_3_Patient2_
Group_3_Patient2_
Group_3_Patient2_
Group_3_Patient2_
